#  Oseltamivir-Resistant Influenza Viruses A (H1N1), Norway, 2007–08

**DOI:** 10.3201/eid1502.081031

**Published:** 2009-02

**Authors:** Siri H. Hauge, Susanne Dudman, Katrine Borgen, Angie Lackenby, Olav Hungnes

**Affiliations:** Norwegian Institute of Public Health, Oslo, Norway (S.H. Hauge, S. Dudman, K. Borgen, O. Hungnes); Health Protection Agency, London, UK (A. Lackenby)

## Abstract

In Norway in January 2008, unprecedented levels of oseltamivir resistance were found in 12 of 16 influenza viruses A (H1N1) tested. To investigate the epidemiologic and clinical characteristics of these viruses, we used sequence analysis to test all available subtype H1N1 viruses from the 2007–08 season for resistance. Questionnaires from physicians provided information on predisposing diseases, oseltamivir use, symptoms, and complications. Clinical data were obtained for 265 patients. In total, 183 (67.3%) of 272 viruses were oseltamivir resistant. Resistance was not associated with prior use of antiviral drugs. Symptoms and hospitalization rates did not differ for patients infected with a resistant or a susceptible virus. Oseltamivir-resistant influenza viruses A (H1N1) did not show diminished capability to spread in the absence of selective pressure. The ability of these viruses to sustain their fitness and spread among persons should be considered when shaping future strategies for treating and preventing seasonal and pandemic influenza.

## Abstract 2

Resistance was not associated with oseltamivir use or more severe disease.

Seasonal influenza, caused by influenza A subtypes H3N2 and H1N1 and influenza B viruses, occurs as annual epidemics. Although vaccination remains the primary measure for prevention, antiviral drugs are available for prevention and treatment of influenza. The influenza virus neuraminidase inhibitors zanamivir and oseltamivir were introduced into clinical practice in various parts of the world from 1999 through 2002 (*1*). Oseltamivir limits replication of both influenza A and B viruses (*1*). In most European countries, neuraminidase inhibitors are not widely used to treat seasonal influenza, but they are being stockpiled in many countries as part of their pandemic influenza preparedness. In Norway, oseltamivir is registered for prophylactic and therapeutic use in persons >1 year of age; however, it is not available without a prescription and it is rarely prescribed (*2*).

Until 2007, resistance against neuraminidase inhibitors was rarely observed (*1*,*3*,*4*). Nevertheless, to better understand the potential for development of resistance against neuraminidase inhibitors, surveillance of antiviral susceptibility in influenza virus in Europe has been ongoing since 2004 (*5*). As part of the World Health Organization (WHO) Global Influenza Surveillance Network, the national influenza centers in Europe submit influenza viruses to the WHO Influenza Collaborating Centre in the United Kingdom each influenza season. Within the framework of the European Surveillance Network for Vigilance against Viral Resistance (VIRGIL), these viruses are also tested for drug susceptibility at the Health Protection Agency in London.

In mid-January 2008, antiviral susceptibility testing (enzyme inhibition assays) of the first shipment of influenza viruses from Norway for the 2007–08 season showed an unusually large proportion (5/7) of influenza viruses A (H1N1) with high-level resistance to oseltamivir. In subsequent days, testing of additional viruses from Norway at the Norwegian national influenza center and at the Health Protection Agency confirmed the high proportion of oseltamivir resistance. This unexpected and unprecedented discovery had possible public health implications of international concern. On January 25, 2008, the Norwegian Institute of Public Health notified WHO of these findings under the International Health Regulations (*6*) and notified the European Commission through the Early Warning and Response System. The Institute also informed hospitals and physicians in Norway about a possible lack of therapeutic effect when treating patients with oseltamivir. By the end of January, oseltamivir-resistant viruses had been reported from several European countries (*7*).

The oseltamivir-resistance trait is caused by a previously described point mutation in the virus neuraminidase gene (histidine to tyrosine at position 275 of the N1 neuraminidase, commonly referred to as H274Y in N2 numbering), which is known to confer high-level resistance to oseltamivir while retaining susceptibility to zanamivir (*8*). Influenza viruses A (H1N1) carrying the H274Y mutation have reduced ability to replicate and transmit efficiently when compared with parental, susceptible virus, but the clinical implications of infection with these viruses have been largely unknown (*9*). Consequently, we undertook studies to determine whether the emergence and spread of the resistant viruses were associated with exposure to oseltamivir, whether resistant viruses would continue to circulate in similar proportions into the epidemic phase of the season, and whether the new resistant viruses differed from their susceptible counterparts in their ability to cause disease. To do so, we tested all influenza viruses A (H1N1) available from the 2007–08 outbreak for oseltamivir susceptibility. We furthermore enhanced surveillance by collecting an extended set of data regarding clinical symptoms, complications, and prior exposure to oseltamivir for all laboratory-verified cases of influenza viruses A (H1N1) infection.

## Methods

The influenza viruses A (H1N1) included in this study were obtained from the sentinel and nonsentinel collaborators as part of routine national virologic influenza surveillance. From all 19 counties, 71 sentinel practices collect samples from patients with influenza-like illness and send them to the national influenza center for diagnostic testing. From all parts of the country, 15 medical microbiology laboratories submit materials containing influenza A or B materials (original specimens, nucleic acid preparations from original specimens, or viral isolates) to the national influenza center for further characterization. Most of these samples originate from primary care clinics; the rest, from hospitals.

Viruses confirmed as influenza A (H1), by either reverse transcription–PCR (RT-PCR) or virus isolation in MDCK cells and subsequent subtyping by immunofluorescence, were included in the study. In-country susceptibility testing was performed by detecting the H274Y mutation by sequence analysis, through either a pyrosequencing assay targeting the single relevant point mutation (*10*) or through full- or partial-length cycle sequencing of the coding region for the viral neuraminidase. These analyses were mostly performed on RNA prepared from the original patient specimen. A large proportion of the isolated viruses were sent to the WHO Collaborative Centre for Influenza Research and Reference in the National Institute of Medical Research, Mill Hill, UK, for further characterization. Within the framework of VIRGIL, these viruses were forwarded to the Health Protection Agency for phenotypic antiviral susceptibility testing and more extensive genotypic analyses. To determine neuraminidase susceptibility, assays to determine the drug concentration that provides 50% inhibition (IC_50_) were performed by using the fluorescent substrate methylumbelliferyl N-acetylneuraminic acid based on the method described by Wetherall et al. (*11*) with minor modifications.

Relative quantitative data on virus shedding, i.e., virus RNA content in the patient specimens, were obtained through a real-time RT-PCR targeting a conserved part of the matrix protein (M1) gene of influenza A virus. Two microliters of nucleic acid prepared from specimens (MagNApure LC Total Nucleic Acid Isolation Kit; Roche Diagnostics, Mannheim, Germany) was added to a 23-μL reaction mixture containing 0.3 μM forward primer M52c (5′-CTT CTA ACC GAG GTC GAA ACG-3′); 0.3 μM reverse primer M149r (5′-CTT GTC TTT AGC CAT TCC ATG AG-3′); 0.15 μM probe M93c (FAM-5′ CCG TCA GGC CCC CTC AAA GCC GA 3′-Black Hole Quencher 1); and 5× QIAGEN OneStep RT-PCR buffer, dNTP mixture, and enzyme mixture according to the manufacturer’s instructions (QIAGEN OneStep RT PCR Kit; QIAGEN, Hilden, Germany). Forward primer and probe sequences were as described by Fouchier et al. (*12*), and the reverse primer was designed by Tom Øystein Jonassen (Akershus University Hospital, Lørenskog, Norway). Reactions were run in a Corbett Rotorgene RG-3000 or RG-6000 thermocycler (Corbett Research Pty Ltd, Sydney, New South Wales, Australia) with the following cycling conditions: reverse trancription for 30 min at 50°C, then 15 min at 95°C, followed by 50 cycles at 95°C for 10 sec, 54°C for 30 sec, and 72°C for 20 sec.

### Participants and Study Design

We included all patients with a diagnosis of influenza virus A (H1N1) infection made by national influenza center during the 2007–08 influenza season. For the 72 patients who received this diagnosis before the end of January 2008, data were collected retrospectively. From February on, data were collected as soon as possible after laboratory confirmation of influenza virus A (H1N1) infection. Structured questionnaires returned from consulting physicians provided auxiliary information about clinical signs and symptoms, complications, predisposing diseases for severe outcome of influenza (diabetes, cardiac disease, lung disease, and immunodeficiency), use of oseltamivir, and influenza vaccination status. If the questionnaire was not returned by mail within 3 weeks, a reminder call was made. When available, relevant clinical information on the original referral sample form was used to supplement the data from the written questionnaire. The consulting physician, usually the primary care physician, was not informed about the result of the susceptibility testing when the information was collected. Information for the first 12 patients infected with a resistant virus was collected from the consulting physicians by telephone.

### Statistical Analysis

Data from the questionnaires and selected laboratory testing outcomes were merged, checked for quality, and analyzed by using Stata version 9.0 (StataCorp LP, College Station, TX, USA). We used the Fisher exact test to compare the proportions of possible confounders among those infected with a resistant and a susceptible virus. To estimate the association between exposure (resistant virus infection) and outcome (subsequent clinical findings and complications), we calculated crude risk ratios (RRs) and 95% confidence intervals (CIs). We used binomial regression to calculate RRs adjusted for possible confounders. For each variable, we used the number of respondents as the denominator, except for predisposing disease, for which missing values were coded as “no.”

## Results

The overall influenza activity in Norway was low in 2007–08 compared with that of previous years. Virologic surveillance showed most influenza virus A and 95% of subtyped viruses to be subtype H1N1 (*13*). From the sentinel practices, the national influenza center received 229 specimens for influenza testing. Of the 108 that were positive for influenza virus, 61 were type A, subtype H1N1, and most of the rest were type B. In total, 297 patients had an influenza virus A (H1N1) infection confirmed by the national influenza center in Norway from week 47 in 2007 until the end of week 20 in 2008. We obtained a resistance profile for 272 of the 297 viruses. We could not determine the resistance profile for the remaining 25 because of low virus content and consequently excluded them from analysis.

**Figure 1:**
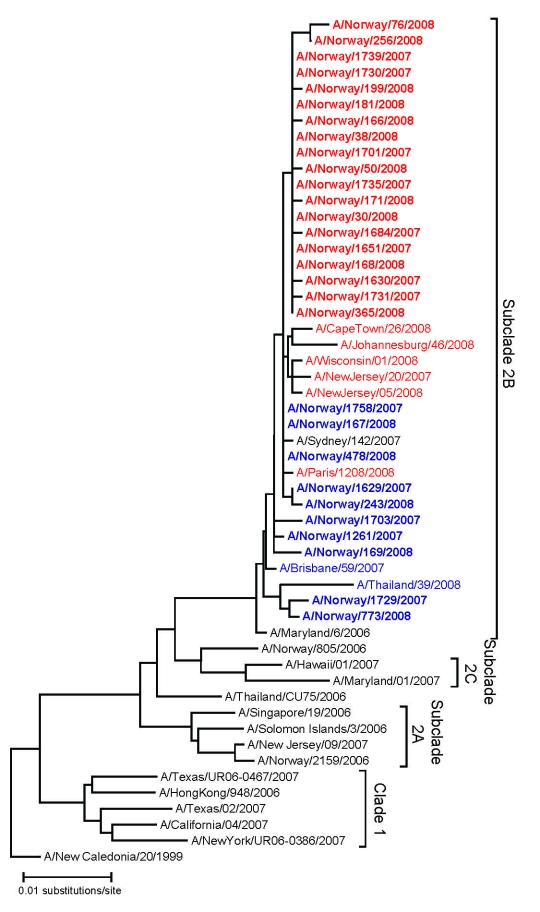
Phylogenetic reconstruction of the H1 genes of influenza viruses A (H1N1) in Norway, 2007–08 season. The analysis was performed on an alignment spanning positions 84–1054 of viral RNA segment 4. Pairwise distances were calculated by using the Kimura 2-parameter model with a transition:transversion ratio of 2.0; the phylogenetic tree was constructed by the neighbor-joining method, as implemented in the programs DNADIST and NEIGHBOR in the PHYLIP package (*14* *15**,*). Published sequences were obtained from the Influenza Sequence Database, Los Alamos National Laboratory (*16*). Boldface indicates viruses from the 2007–08 influenza season in Norway; red indicates oseltamivir-resistant viruses; blue, susceptible viruses. New sequences presented in this analysis have been deposited in GenBank (accession nos. CY036664–CY036694).

A total of 196 viral isolates were available (133 carried the resistance mutation); of these, 113 (79 with the resistant genotype) were reference tested by the VIRGIL laboratory. Phenotypic and genotypic reference analysis results agreed completely with the in-country genotypic testing results; all mutant viruses showed large reductions in susceptibility to oseltamivir when compared with non-H274Y viruses (IC_50_ 260–2,161 nM, mean 673 nM, for the 274Y mutant and 0.4–5.6 nM, mean 2.6 nM, for the nonmutant viruses). No evidence of mixed genotype or phenotype was observed. In phylogenetic analysis of the H1 gene, all viruses tested grouped together in subclade 2B (Figure 1). In the phylogenetic tree, the resistant viruses from Norway all formed a single branch that was distinct, but closely related, to the susceptible viruses from Norway.

**Figure 2:**
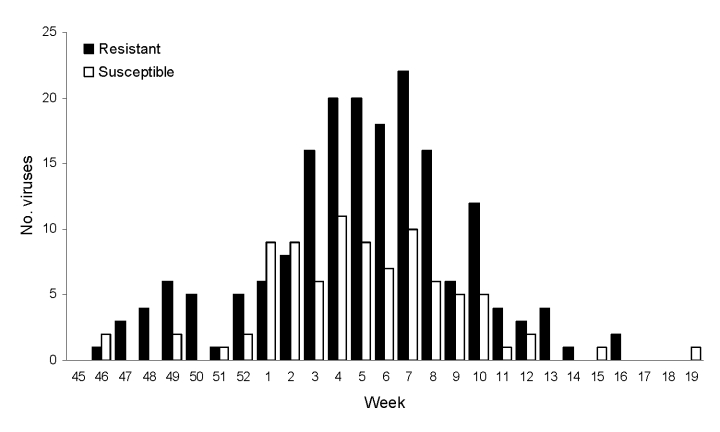
Oseltamivir-resistant (n = 183) and oseltamivir-susceptible (n = 89) influenza viruses A (H1N1) in the 2007–08 influenza season in Norway, by week of sampling.

**Figure 3:**
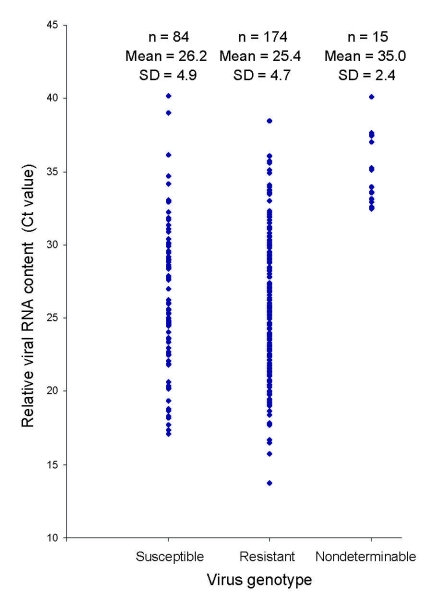
Comparison of virus shedding, measured as relative viral RNA content, in respiratory specimens taken from patients infected with oseltamivir-susceptible and oseltamivir-resistant influenza viruses A (H1N1), respectively, during the 2007–08 influenza season in Norway. Viral RNA content is expressed as the reverse-transcription–PCR cycle number (Ct) during which the fluorescence threshold was exceeded.

**Figure 4:**
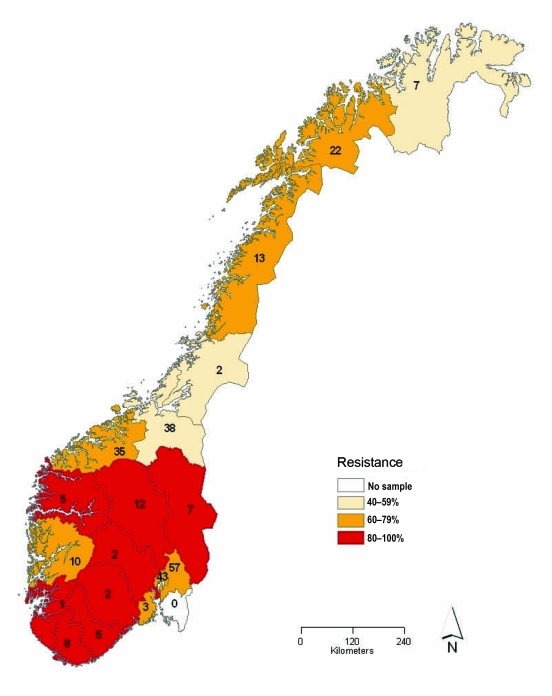
Proportion of oseltamivir-resistant influenza viruses A (H1N1) in the 2007–08 influenza season in Norway, by county of sampling. The total number of samples analyzed for each county is given inside each county.

Of the 272 influenza viruses A (H1N1), 183 (67.3%) were oseltamivir resistant (Table 1). The proportion of resistant viruses did not differ between samples from sentinel 67.9% (38/56) and nonsentinel 67.1% (145/216) practices and persisted throughout the season (Figure 2). No difference in virus shedding, as quantified by real-time RT-PCR of available patient specimens, was observed between susceptible and resistant viruses (Figure 3). From the original sample form, we obtained demographic information for all 272 patients. Returned questionnaires provided information for 265 patients (97.4%), but the response rate on individual questions varied. Of the 272 patients infected with influenza viruses A (H1N1), 132 (48.5%) were male (Table 1), and slightly more than half (50.7%) were 29–64 years of age (median 27 years, range 2 months–71 years); median ages of those infected with a resistant and a susceptible virus were 31 and 21 years, respectively. The highest proportion of resistant virus infection was found for those 25–59 years of age (102/138, 73.9%) and differed significantly from the proportion for only those 5–14 years of age (25/45, 55.6%) (Fisher exact p = 0.03). We obtained influenza viruses A (H1N1) from 18/19 counties (Figure 4).The oseltamivir resistance proportion was >80% in 8 counties in southern Norway, compared with 63.5% in the rest of the country (Fisher exact p = 0.001).

**Table 1:** Proportion of oseltamivir-resistant and oseltamivir-susceptible influenza viruses A (H1N1), 2007–08 influenza season, Norway

Sample source	Total no. samples	No. (%) resistant samples	No. (%) susceptible samples
All	272	183 (67.3)	89 (32.7)
Type of practice			
Sentinel	56	38 (67.8)	18 (32.1)
Nonsentinel	216	145 (67.1)	71 (32.9)
Patient gender			
Male	132	85 (64.4)	47 (35.6)
Female	140	98 (70.0)	42 (30.0)
Patient age group, y			
0–4	45	27 (60.0)	18 (40.0)
5–14	45	25 (55.6)	20 (44.4)
15–24	31	20 (64.5)	11 (35.5)
25–59	138	102 (73.9)	36 (26.1)
60–99	13	9 (69.2)	4 (30.8)
Patient with predisposing disease			
Diabetes	10	9 (90.0)	1 (10.0)
Lung disease	11	8 (72.7)	3 (27.3)
Cardiac disease	5	2 (40.0)	3 (60.0)
Immunodeficiency	5	3 (60.0)	2 (40.0)
Any	27	20 (74.1)	7 (25.9)

Information about use of antiviral drugs was obtained for 237 patients. No patients had received antiviral treatment in the 14 days before the onset of symptoms, and none had been in close contact with others known to have used antiviral drugs. Oseltamivir was received after sampling by 7 patients, 5 of whom were infected with an oseltamivir-resistant virus. Of 225 patients, 9 had traveled abroad in the week before symptom onset; 4 were infected with a resistant virus. Of all 272 patients, 2 had been vaccinated against influenza and were both infected with a resistant virus.

We received information about predisposing disease for 213 patients. Having a predisposing disease more than doubled the risk for complications (RR 2.5, 95% CI 1.2–5.4) but was not clearly associated with being infected with a resistant virus (RR 1.4, 95% CI 0.6–3.2). Information about clinical symptoms was obtained for 252/272 patients; most frequently reported were fever (229/242) and dry cough (182/218). Resistant virus infection was not associated with any particular symptom (Table 2). Of 241 patients, 58 (24.1%) had >1 complications recorded, but no difference was observed between those infected with a resistant virus and those infected with a susceptible virus (Table 2). Bronchitis and pneumonia were the most frequent complications, reported for 22 and 17 patients, respectively. The age of the 17 patients who had pneumonia ranged from 8 months to 65 years (mean 29 years): 2 (12.5%) were 0–4 years of age, 5 (31.3%) were 5–14 years of age, 2 (12.5%) were 15–24 years of age, 4 (25.0%) were 25–59 years of age, and 3 (18.8%) were >59 years of age. Of the 17 patients with pneumonia, 15 were infected with a resistant virus. The attack rates of pneumonia and of sinusitis were higher for those infected with a resistant virus than for those infected with a susceptible virus, although the risk ratios were not statistically significant after adjusting for age, gender, and predisposing disease (pneumonia RR 3.2, 95% CI 0.7–13.7; sinusitis RR 1.7, 95% CI 0.4–7.5) (Table 2). Of 264 patients, 45 had been hospitalized, 28 and 17 infected with a resistant and a susceptible virus, respectively. No deaths were reported for patients included in the study.

**Table 2:** Reported associations for patients infected with oseltamivir-resistant or oseltamivir-susceptible influenza virus A (H1N1), 2007–08 influenza season, Norway

Association	Resistant, n = 183			Susceptible, n = 89			Crude associations		Adjusted associations*
	Attack rate, %	No. responses		Attack rate, %	No. responses		Risk ratio (95% CI)†		Risk ratio (95% CI)
Sign or symptom									
Productive cough	38.4	125		31.9	47		1.2 (0.8–1.9)		
Fever	94.4	162		95.0	80		1.0 (0.9–1.1)		
Myalgia	72.9	129		73.3	60		1.0 (0.8–1.2)		
Dry cough	82.1	145		86.3	73		1.0 (0.8–1.1)		
Headache	63.4	131		67.2	58		0.9 (0.8–1.2)		
Sore throat	57.5	134		67.2	58		0.9 (0.7–1.1)		
Runny nose	62.2	127		66.1	56		0.9 (0.8–1.2)		
Complication									
Pneumonia	9.2	153		2.9	69		3.2 (0.7–13.5)		3.2 (0.7–13.7)
Sinusitis	6.2	145		3.0	67		2.1 (0.5–9.4)		1.7 (0.4–7.5)
Otitis media	4.8	145		4.4	69		1.1 (0.3–4.2)		1.3 (0.4–4.8)
Bronchitis	8.7	149		11.8	76		0.7 (0.3–1.7)		0.8 (0.4–1.8)
Any	24.4	164		22.1	77		1.1 (0.7–1.8)		1.1 (0.7–1.8)
Hospitalization	15.8	177		19.5	87		0.8 (0.5–1.4)		0.8 (0.5–1.3)

*Risk ratio adjusted for age, gender, and predisposing disease. †CI, confidence interval.

## Discussion

During the 2007–08 influenza season in the Northern Hemisphere, widespread circulation of oseltamivir-resistant influenza viruses A (H1N1) was observed. Percentage of resistant viruses circulating in different countries varied markedly; the highest proportion reported worldwide (67%) was in Norway (*17**,**18*).

Our study did not show any association between oseltamivir use in Norway and emergence of the oseltamivir-resistant influenza viruses A (H1N1). Because only a minority of influenza cases are laboratory confirmed, oseltamivir use in nonsampled persons could have contributed to the development of resistance. However, for this suggestion to be plausible, use of oseltamivir would have to be widespread to exert substantial selective pressure on the viruses. Sales of oseltamivir in Norway have been low: 699 5-day regimens (0.15/1,000 population) were sold in 2004; 66,249 (14.4/1,000 population) in 2005; 33,573 (7.3/1,000 population) in 2006; and 4,686 (1.0/1,000 population) in 2007 (*2*). In countries where oseltamivir use has been high, e.g., Japan, the proportion of oseltamivir-resistant influenza viruses A (H1N1) reported during the 2007–08 season was low (*18*). Because influenza strains from Norway were genetically similar to resistant viruses that appeared just as early in several other European countries (A. Hay, pers. comm.), we consider it unlikely that the resistant variant originated in Norway. Conceivably, the initial emergence of a resistant virus could be associated with oseltamivir use elsewhere. Our data indicate that the viruses carrying this resistance mutation are fully capable of persistence and spread in the absence of selective pressure.

In Norway, the initially high proportion of resistant influenza viruses A (H1N1) was maintained throughout the entire 2007–08 influenza season; countrywide, 2 of 3 viruses were resistant. The reason for this exceptionally high resistance proportion is unknown. However, it likely reflects the proportion of resistant viruses introduced into Norway in the fall of 2007. Globally, the proportion of resistant influenza viruses A (H1N1) reported is highly variable between the different countries for which data are available (*18*). This large variation, apparently in the absence of oseltamivir selective pressure, suggests that a high level of randomness determined the frequency of resistance. In temperate countries, the influenza viruses are reintroduced each autumn after the absence of influenza during the summer. If only a limited number of viruses are reintroduced into each country and initiate virus circulation and outbreaks, the result will be considerable random variation in virus variant proportions between the different countries. Consistent with this result, almost all the characterized influenza viruses A (H1N1) in Norway could be assembled into a small number of genetically discernible groups (Figure 1). We propose that such randomness in virus introductions may be sufficient to explain the differences in the proportions of resistant viruses between the countries.

Conceivably, a difference in the antigenic characteristics of the resistant and susceptible viruses could have favored one virus over the other in the face of host population immunity. Such differences might contribute to different relative effect of the 2 viruses in different populations (e.g., countries) or subpopulations (e.g., age groups). However, the resistant and susceptible viruses were closely related and were not distinguishable in hemagglutination inhibition tests (*19*).

Overall, the observed clinical manifestations associated with influenza viruses A (H1N1) in this study were as expected for seasonal influenza. No differences were noted for virus shedding, primary symptoms, or overall complication and hospitalization rates caused by oseltamivir-resistant and -susceptible viruses. We did find, although not a statistically significant finding, that patients infected with a resistant virus appeared to be more likely than those infected with a susceptible virus to have pneumonia or sinusitis. Patients with more severe illness may be more likely to be sampled; however, the resistance pattern of the virus was not known by the physician at the time of sampling and reporting. We therefore believe that these findings are not influenced by selection bias. Adjusting for possible confounders (age, sex, and predisposing disease) did not change the results. Because of our limited sample size, the precision of our estimates is low, but they do indicate findings that warrant further investigation. Our data will also be analyzed with data from other European countries, and the findings may strengthen the conclusions about the clinical implications of oseltamivir-resistant influenza viruses A (H1N1) .

The future effect of resistant influenza viruses A (H1N1) is unpredictable. In Europe, the H1N1 subtype was predominant during the 2007–08 influenza season and, according to historical patterns, is unlikely to predominate during the 2008–09 influenza season. In the following 2008–09 season in the Northern Hemisphere, influenza viruses A (H1N1) may well predominate in areas where they had not recently been present in large numbers. Early reporting from the Southern Hemisphere 2008 influenza season indicates that detection of influenza virus A (H1N1) is low (*20*). However, in South Africa, oseltamivir resistance has been detected in 100% of influenza viruses A (H1N1) tested (*21*).

Whether oseltamivir-resistant viruses will persist beyond 2008 depends on several factors. First, their persistence will depend on the prevalence of resistant viruses in the populations that are the source of global influenza spread. Countries in East and Southeast Asia have been proposed as the most likely source for global dissemination of new influenza virus variants (*22*). The prevalence of resistant influenza viruses A (H1N1) in this region may therefore be more likely to influence future occurrence of these viruses than the prevalence in Europe; resistance monitoring thus needs to be global. Second, changes in recent influenza viruses A (H1N1) may have provided a genetic background that permits H274Y mutants to replicate and transmit. Previous studies have concluded that resistant viruses are less pathogenic and less transmissible than their susceptible counterparts (*9**,**23*). In contrast, however, reverse genetics–derived mutants (A/WSN/33 or PR8 backbone) had the same phenotype as wild type viruses in vitro and in vivo (*24**,**25*). A recent study on the enzymatic properties of the N1 neuraminidase of the resistant viruses from the 2007–08 season suggested some genetic background changes that could potentially be involved (*26*).

As long as such a postulated permissive genetic background is common, resistant mutants may arise anew in purely oseltamivir-susceptible influenza virus A (H1N1) populations. Identification of such predisposing genetic traits and monitoring of their occurrence in influenza viruses A (H1N1) and other influenza viruses should continue.

Similar resistance can arise in viruses other than the current human influenza viruses A (H1N1). Resistance in a more virulent influenza virus can have serious public health implications because of fewer therapeutic and prophylactic options, which may result in more persons being affected by influenza and more severe illness and death in those who become infected. Oseltamivir is a prime option for influenza treatment and prophylaxis and forms a substantial part of pandemic preparedness in many countries. The prevalence of oseltamivir-resistant viruses reported in Europe throughout the 2007–08 influenza season clearly shows that this resistant mutation is stable and that these viruses sustain their fitness and ability to spread among persons. These findings should be taken into consideration when shaping future strategies for treating and preventing seasonal and pandemic influenza.

## References

[1] McKimm-Breschkin J, Trivedi T, Hampson A, Hay A, Klimov A, Tashiro M, Neuraminidase sequence analysis and susceptibilities of influenza virus clinical isolates to zanamivir and oseltamivir. Antimicrob Agents Chemother. 2003;47:2264–72. 10.1128/AAC.47.7.2264-2272.2003 12821478 PMC161875

[2] Aavitsland P, Hauge S, Borgen K. Rare usage of oseltamivir in Norway prior to emergence of oseltamivir resistant influenza A(H1N1) virus in the 2007–2008 season. 2008 International Conference on Emerging Infectious Disases; 2008 Mar 16–19; Atlanta. Addendum 11.

[3] Escuret V, Frobert E, Bouscambert-Duchamp M, Sabatier M, Grog I, Valette M, Detection of human influenza A (H1N1) and B strains with reduced sensitivity to neuraminidase inhibitors. J Clin Virol. 2008;41:25–8. 10.1016/j.jcv.2007.10.019 18055253

[4] Monto AS, McKimm-Breschkin JL, Macken C, Hampson AW, Hay A, Klimov A, Detection of influenza viruses resistant to neuraminidase inhibitors in global surveillance during the first 3 years of their use. Antimicrob Agents Chemother. 2006;50:2395–402. 10.1128/AAC.01339-05 16801417 PMC1489772

[5] Zambon M, Hayden FG. Position statement: global neuraminidase inhibitor susceptibility network. Antiviral Res. 2001;49:147–56. 10.1016/S0166-3542(01)00124-3 11428241

[6] World Health Organization. International Health Regulations (IHR) 2005, 2nd ed. [cited 2008 December 8]. Available from http://www.who.int/csr/ihr/IHR_2005_en.pdf

[7] Lackenby A, Hungnes O, Dudman SG, Meijer A, Paget WJ, Hay A, Emergence of resistance to oseltamivir among influenza A(H1N1) viruses in Europe. Euro Surveill. 2008;13. pii: 8026. 10.2807/ese.13.05.08026-en 18445375

[8] Mishin VP, Hayden FG, Gubareva LV. Susceptibilities of antiviral-resistant influenza viruses to novel neuraminidase inhibitors. Antimicrob Agents Chemother. 2005;49:4515–20. 10.1128/AAC.49.11.4515-4520.2005 16251290 PMC1280118

[9] Ives JA, Carr JA, Mendel DB, Tai CY, Lambkin R, Kelly L, The H274Y mutation in the influenza A/H1N1 neuraminidase active site following oseltamivir phosphate treatment leaves virus severely compromised both in vitro and in vivo. Antiviral Res. 2002;55:307–17. 10.1016/S0166-3542(02)00053-0 12103431

[10] Lackenby A, Democratis J, Siqueira M, Zambon M. Rapid quantitation of neuraminidase inhibitor drug resistance in influenza virus quasispecies. Antivir Ther. 2008;809–20. 18839782

[11] Wetherall NT, Trivedi T, Zeller J, Hodges-Savola C, McKimm-Breschkin JL, Zambon M, Evaluation of neuraminidase enzyme assays using different substrates to measure susceptibility of influenza virus clinical isolates to neuraminidase inhibitors: report of the neuraminidase inhibitor susceptibility network. J Clin Microbiol. 2003;41:742–50. 10.1128/JCM.41.2.742-750.2003 12574276 PMC149673

[12] Fouchier RA, Bestebroer TM, Herfst S, Van Der Kemp L, Rimmelzwaan GF, Osterhaus AD. Detection of influenza A viruses from different species by PCR amplification of conserved sequences in the matrix gene. J Clin Microbiol. 2000;38:4096–101. 11060074 10.1128/jcm.38.11.4096-4101.2000 PMC87547

[13] Norwegian Institute of Public Health. The 2007/2008 influenza season in Norway [cited 2008 May 28]. Available from http://www.fhi.no/eway/default.aspx?pid=238&trg=MainLeft_5895&MainArea_5811=5895:0:15,2820:1:0:0:0:0&MainLeft_5895=5825:66508:1:5896:3:0:0

[14] Felsenstein J. PHYLIP (phylogeny inference package) version 3.2. Cladistics. 1989;5:164–6.

[15] Felsenstein J. PHYLIP (phylogeny inference package) version 3.5c. Seattle: Department of Genetics, University of Washington; 1993.

[16] Macken C, Lu H, Goodman J, Boykin L. The value of a database in surveillance and vaccine selection. In: Osterhaus ADME, Cox N, Hampson AW, editors. Options for the control of influenza IV. Amsterdam: Elsevier Science; 2001. p. 103–6.

[17] European Centre for Disease Prevention and Control. Antivirals and antiviral resistance influenza [cited 2008 May 28]. Available from http://ecdc.europa.eu/en/Health_topics/influenza/antivirals_table.aspx

[18] World Health Organization. Influenza A(H1N1) virus resistance to oseltamivir—last quarter 2007 to 5 May 2008 [cited 2008 May 5]. Available from http://www.who.int/csr/disease/influenza/H1N1ResistanceWeb20080505.pdf

[19] World Health Organization. Recommended composition of influenza virus vaccines for use in the 2009 southern hemisphere influenza season [cited 2008 October 5]. Available from http://www.who.int/entity/csr/disease/influenza/200809Recommendation.pdf 18846716

[20] World Health Organization. Seasonal influenza activity in the world, 2008 [cited 2008 July 24]. Available from http://www.who.int/csr/disease/influenza/update/en/index.html

[21] World Health Organization. Influenza A(H1N1) virus resistance to oseltamivir [cited 2008 July 18]. Available from http://www.who.int/csr/disease/influenza/h1n1_table/en/index.html

[22] Russell CA, Jones TC, Barr IG, Cox NJ, Garten RJ, Gregory V, The global circulation of seasonal influenza A (H3N2) viruses. Science. 2008;320:340–6. 10.1126/science.1154137 18420927

[23] Herlocher ML, Carr J, Ives J, Elias S, Truscon R, Roberts N, Influenza virus carrying an R292K mutation in the neuraminidase gene is not transmitted in ferrets. Antiviral Res. 2002;54:99–111. 10.1016/S0166-3542(01)00214-5 12062395

[24] Abed Y, Baz M, Boivin G. Impact of neuraminidase mutations conferring influenza resistance to neuraminidase inhibitors in the N1 and N2 genetic backgrounds. Antivir Ther. 2006;11:971–6. 17302366

[25] Yen HL, Ilyushina NA, Salomon R, Hoffmann E, Webster RG, Govorkova EA. Neuraminidase inhibitor-resistant recombinant A/Vietnam/1203/04 (H5N1) influenza viruses retain their replication efficiency and pathogenicity in vitro and in vivo. J Virol. 2007;81:12418–26. 10.1128/JVI.01067-07 17855542 PMC2169015

[26] Rameix-Welti MA, Enouf V, Cuvelier F, Jeannin P, van der Werf S. Enzymatic properties of the neuraminidase of seasonal H1N1 influenza viruses provide insights for the emergence of natural resistance to oseltamivir. PLoS Pathog. 2008;4:e1000103. 10.1371/journal.ppat.1000103 18654625 PMC2453323

